# Oral Microbiota Taxa and Pri-miRNA Expression in Bipolar Disorder: A Case–Control Study

**DOI:** 10.3390/biom15101355

**Published:** 2025-09-24

**Authors:** Diego Primavera, Mauro Giovanni Carta, Massimo Tusconi, Goce Kalcev, Laura Atzori, Caterina Ferreli, Rober Romero Ramirez, Letizia Peddio, Cinzia Casu, Sara Fais, Germano Orrù, Alessandra Scano

**Affiliations:** 1 Department of Medical Sciences and Public Health, University of Cagliari, Monserrato Blocco I (CA), 09042 Cagliari, Italy; diego.primavera@unica.it (D.P.); maurogcarta@gmail.com (M.G.C.); 2 PhD Program in Tropical Medicine, Universidad Popular del Cesar, Valledupar 200001, Colombia; 3 Department of Nursing, Universidad Popular del Cesar, Valledupar 200001, Colombia; 4 Center for Liaison Psychiatry and Psychosomatics, University Hospital of Cagliari, 09042 Cagliari, Italy; gocekalcev@yahoo.com; 5 Dermatology Clinic, Department of Medical Sciences and Public Health, University of Cagliari, 09042 Cagliari, Italy; atzoril@unica.it (L.A.); ferreli@unica.it (C.F.); 6 Department of Rectory, Popular University of Cesar, Valledupar 200001, Colombia; roberromero@unicesar.edu.co; 7 Department of Surgical Sciences, University of Cagliari, Cittadella Universitaria, 09042 Cagliari, Italy; l.peddio6@studenti.unica.it (L.P.); sarafais79@gmail.com (S.F.); orru@unica.it (G.O.); 8 Oral Biotechnology Laboratory, Department of Surgical Science, University of Cagliari, 09124 Cagliari, Italy; cinzia.casu2@unica.it; 9 National Research Council of Italy, ISPA-CNR, 07100 Sassari, Italy

**Keywords:** bipolar disorders, oal microbiome, pri-miRNA 155, pri-miRNA 146a, biomarkers

## Abstract

**Background/Objectives:** Emerging evidence suggests a role for oral microbiota in mood disorders, particularly bipolar disorder (BD), complementing established links between gut dysbiosis and psychiatric symptoms. This study investigates the composition of oral microbial taxa and the expression of inflammation-related pri-miRNAs (146a and 155) in individuals with BD, aiming to explore their potential as biomarkers in the oral–gut–brain axis. **Methods:** A matched case–control design was implemented, recruiting 25 BD patients and 46 controls matched by age and sex. Salivary samples were collected, and microbial profiling was conducted via real-time qPCR targeting major bacterial phyla and genera. Pri-miRNA 146a and 155 expression was evaluated through RT-qPCR using validated primers. Statistical comparisons between groups were performed using Fisher’s exact test and non-parametric tests for continuous variables. **Results:** Microbial analysis revealed significant reductions (*p* < 0.01) in α-Proteobacteria, γ-Proteobacteria, and Actinobacteria in BD patients versus controls. A shift toward a higher Firmicutes/Bacteroidetes ratio was observed in the BD cohort, suggesting differences in the oral biotic status between the two groups. However, pri-miRNA 146a and 155 expression levels did not differ significantly between the groups and exhibited high inter-individual variability. **Conclusions:** The findings indicate that oral microbiota composition differs in BD patients, potentially influencing systemic homeostasis through interactions with gut microbial communities and SCFA pathways. These findings should be interpreted as preliminary and hypothesis-generating given the modest sample size. While pri-miRNAs 146a and 155 did not distinguish BD status, the observed microbial taxa alterations should be regarded as exploratory and hypothesis-generating. Larger, longitudinal studies are required to clarify their potential role in BD pathogenesis and risk assessment.

## 1. Introduction

Recently, in the field of mood disorders (MDs), some studies have highlighted oral health as a key factor associated with these illnesses [1,2,3,4,5,6], even though most focus is usually on the gut microbiome’s effect on mood [6,7,8,9,10,11,12,13]. Based on these considerations and hypotheses, two main oral factors should be involved in the modulation of mood disorders: the microbiome profile and the stress pattern level in the oral mucosa [1,13,14,15,16,17]. These factors are closely connected because an imbalance in oral microflora can harm the tissues underneath, for example, by creating harmful substances like proteases or various bacterial byproducts [18,19,20]; on the other hand, tissue inflammation can modify the composition of the microbiota and its pathogenicity profile. Considering some aspects of mood (MD) disorder modulation, Levert-Levitet et al. found a link between oral microbiota and veterans with post-traumatic stress disorder (PTSD) [21]. Li et al. highlighted an association between oral microflora and bipolar disorders [22]. The authors believe that the way bipolar disorder (BD) develops is connected to the types of bacteria found in the mouth, based on their Mendelian randomisation data [23,24]; in fact, the processes that lead to BD I and BD II can vary greatly depending on the specific types of microbes associated with these different BD subtypes [25,26,27,28,29,30,31,32,33,34,35,36,37,38]. At the same time, these researchers and others note that the differences in bacteria among these patients are closely linked to their habits, medications, and other factors, making it difficult to use a simple microbiome profile for diagnosis [39,40,41,42,43]. In this context, we could identify a secondary biological parameter to complement the microbial profile, thereby enhancing the prognostic value for BD [44,45,46,47,48]. An interesting candidate could be pri-microRNAs (pri-miRNAs): they are medium-length RNA molecules that undergo processing to become mature miRNAs, in other words, small RNA oligos that control gene expression [49,50]. They participate in gene silencing, and different main cellular functions are regulated by miRNA. It is well recognised that miRNAs control a wide range of important cellular processes, including growth, differentiation, metabolism, and development; are involved in some pathological biological pathways, such as oncogenesis, infective processes, or tissue oxidative stress; and are key components in other gene regulation networks [12,51,52]. In these contexts, miRNAs have been linked to numerous human disorders and have been investigated as plausible candidates for involvement in the majority of biological processes [53]. Different authors [54,55,56,57] emphasise the connection between salivary/oral miRNA and various human disorders, including some that are linked to their pri-miRNA precursors [58,59]. However, the literature does not adequately represent the relationship between miRNA (or pri-miRNA) and oral microbiota in mood disorders. This work is aimed at investigating the connection between oral microbiota and two primary miRNAs involved in the oxidative status of oral mucosa and subjects affected by bipolar disorder. For this reason, we have investigated the function of miRNAs 146a and 155, which have been shown to modulate different biochemical processes in humans, including oxidative stress and inflammation levels. These regulatory molecules are strictly bound to microbiota performance [60,61,62,63,64,65]. In particular, Zhao et al. highlighted the relationship with miRNA 155 and gut microbiota, and the oral delivery of the miR-155 antagonist restored a commensal-rich bacteria microbiota and thus protected cardiac function in a mouse model [65,66,67,68,69]. Considering various articles, a deficient miRNA 146a level in the gut microbiota appears to have a protective function against pathogens [62]. Furthermore, these miRNAs may alter important processes, like breaking down lysine and lengthening fatty acids, which help control certain gut bacteria, like *Prevotella* spp. [70], a genus also crucial in the oral microbiota [71,72]. So far, there has not been much research on mood disorders or how they relate to inflammation in the mouth [73,74,75,76,77]. However, a study by Sancassiani et al. [36], found that when analysing breath samples from healthy people and those with BD, there were higher levels of volatile sulphur compounds (VSCs) in the mouth of BD patients compared with healthy individuals [78,79,80,81]. An increase in VSC levels is associated with a dysbiotic status in the oral microbiome and may also be linked to various systemic diseases and oral tissue inflammation patterns [82,83,84]. In this exploratory case–control study, we hypothesised that patients with BD would show alterations in oral microbial taxa and differences in inflammation-related pri-miRNAs (146a and 155), potentially reflecting oral–gut–brain axis dysregulation.

## 2. Materials and Methods

### 2.1. Patients and Study Design

The design of our study was matched case–control. According to the DSM-5 criteria, the cases were those who had been diagnosed with bipolar disorder, and the controls were chosen among people who had not been diagnosed with the disorder. These subjects have been described in our previously published work [36]. Briefly, in order to compare the Microbiome Taxa and miRNAs profile of the two groups, the control group was created using block randomisation matched for sex and age (± 5 years) to ensure comparability between cases and controls. Another analysis was conducted by treating individuals who did not have a diagnosis of bipolar disorder but scored positively on the Mood Disorder Questionnaire as cases and those who scored negatively on the Mood Disorder Questionnaire as controls [85]. The inclusion criterion was patients aged between 20 and 69 years without any exclusion by sex. Participants were gathered and assessed over three days at Cagliari University Hospital. The subjects completed the MDQ, underwent a psychiatric interview, and provided a general anamnesis after being fully informed about the research study and signing the informed consent form. Block randomisation matching was used to choose the control group, guaranteeing that each case was matched with two controls who were within ±5 years of age and of the same sex. For every case, a control-matching cell that included all eligible people who had not been diagnosed with bipolar disorder was created. For every case, two controls were chosen at random from these matched cells. To preserve independence across matched sets, controls were disqualified from further eligibility after they were chosen. Overall, in this study, we evaluated a total of 25 patients (No. 10 males, mean age: 52.40; No. 15 females, mean age: 58.53) and 46 controls (No. 16 males, mean age: 52.18; No. 30 females, mean age: 63.16). This study was conceived as a pilot, exploratory matched case–control investigation designed to screen for large between-group differences rather than to deliver definitive estimates. No a priori power calculation was performed; therefore, statistical power is limited, and the generalisability of the effect estimates is restricted. Approval for the study was granted by the Ethical Committee of the Institutional Review Board of the University Hospital of Cagliari, Italy (authorisation signed on 11 July 2022, with reference number NP/2022/2893; amendments approved on 6 November 2023 and 15 November 2023). All included subjects provided written informed consent. The 1964 Helsinki Declaration’s standards were followed in the conduct of this investigation. Following a thorough explanation of the study’s objectives, methods, and data protection, all study participants signed a written informed consent form. They were also made aware that the study could end at any time.

### 2.2. Sampling and Nucleic Acid Extraction

For each subject recruited in this study, we collected saliva samples by using a method already described [85]. The patients rinsed their mouth with about 200 µL of commercial mineral water (Rocchetta^®^, Roma, Italy) twice. After 20 min, we collected about 2 mL of saliva in a 2 mL RNase- and DNase-free Eppendorf^®^ tube (Merck KgaA, Darmstadt, Germany). Saliva samples for DNA extraction were used as such, while in tubes for RNA extraction, 100 µL of RNAlater^®^ solution was added immediately, and all samples were then frozen at −80 °C until nucleic acid extraction. DNA and RNA were extracted by using (i) the Bosphore Genomic DNA Extraction Spin Kit v2 and (ii) the Bosphore RNA Extraction Spin Kit, manufactured by Anatolia Geneworks (Istanbul, Turkey), following the kits’ work instructions for each extraction type. The samples were then measured to assess the total DNA/RNA quantity and purity by using a Nanodrop Spectrophotometer (Thermo Fisher Scientific—Waltham, MA, USA).

### 2.3. Microbial Group Evaluation

We examined the main types of oral microbial taxa/genera by using a modified method described by Bachetti et al. [86], which included two new target bacterial groups, *Fusobacterium* spp. and *Candida* spp., as listed in Table 1. We then evaluated all oligos in silico, employing various approaches.

An in silico evaluation of the oligonucleotide sequence was performed by using the Basic Local Alignment Search Tool program (BLAST version 2.17.0), https://blast.ncbi.nlm.nih.gov/Blast.cgi (accessed on 17 July 2025).

The Oligo version 7 (MedProbe, Oslo, Norway) calculator and the DNA-melt program, available at https://www.unafold.org/hybrid2.php (accessed on 17 July 2025) were used to assess the theoretical melting temperatures (Tms) of all primers used in this work, as well as the formation of potential oligonucleotide dimers and self-complementarity.

The DNA folding analysis of the amplicons was assessed using a DNA fold program: https://www.unafold.org/ (accessed on 17 July 2025). This evaluation was performed to ensure that the primer matching zone was free of amplicon loops, which could cause thermodynamic problems, such as varying PCR efficiency for different taxa [87]. In each mentioned program, we used the same physical oligo conditions, setting [DNA] at 10^−7^ M, 50 mM [NaCl], and 3 mM [MgCl].

This study employed a targeted qPCR-based approach designed to quantify the relative abundance of selected bacterial taxa. As such, the data do not permit calculation of ecological diversity indices (e.g., Shannon and Simpson) or ordination analyses (PCA/PCoA), which require broader sequencing-based datasets. This methodological choice was intentional to focus on specific phyla and genera previously implicated in psychiatric conditions.

### 2.4. Real-Time PCR Reaction

Real-time PCR reaction was performed to quantify the total bacterial taxa/genera in DNA extracts. Real-time qPCR profiling was performed using a CFX-96 apparatus (Bio-Rad Laboratories, Hercules, CA, USA) and SYBR Premix Ex Taq Kit (TaKaRa-Clontech ^®^, Kusatsu, Japan), according to the manufacturers’ instructions. The final volume of 0.02 mL contained Premix Ex Taq (1X), 1× SYBR Green (10,000×), 0.22 μM of each primer, and 1 to 10 ng of DNA extract. qPCR thermal profiles consisted of a denaturation step at 95 °C for 30 s, followed by 40 cycles of 5 s at 95 °C, 30 s at 60 °C, and 20 s at 80 °C. For the quantification of different phyla, universal primers were used as positive controls for each sample (Table 1). As a positive reaction control for each sample, we used the universal primers as described in Table 1. qPCR thermal profiles consisted of a denaturation step at 95 °C for 30 s, followed by 40 cycles of 5 s at 95 °C, 30 s at 60 °C, and 20 s at 80 °C. Fluorescence was detected at the end of the 80 °C segment in the PCR step. The bacterial concentration was obtained by interpolating the threshold cycle value of the sample with the standard curve, which we obtained using a series of *Escherichia coli* cell dilutions ranging from 5 × 10^2^ to 1 × 10^8^ bacterial genomes/µL.

All qPCR products were validated by melting-curve analysis, which consistently showed single, sharp peaks, and by comparison with the expected amplicon sizes reported in Table 1. No-template controls remained negative in all runs, confirming amplification specificity.

For each taxonomic group, the concentration was then expressed as genomes/µL corresponding to a suspension of 100 ng DNA/mL, using the following formula:

C_100µg_ = C_pcr_ × 100/[DNA]

where C_100µg_ represents the genomes/µL for the considered bacterial group, contained in a suspension of 100 µg/µL of total DNA. G_pcr_ represents the genomes/ul evaluated with qPCR and contained in the sample. [DNA] = DNA concentration in µg/µL of the sample measured with the Nanodrop instrument version 8.

The percentage concentration for each phylum is calculated as follows:

G% = C_100ng_ × 100/∑_bg_

where C_100ng_ = represents the genomes in 100 ng/mL of total DNA calculated with the previous formula. ∑_Bg_ = sum of the concentration of genomes/100 ng [DNA] of all bacteria in the analysed group.

The qPCRs showed an efficiency range from 0.95 to 0.98.

### 2.5. Pri-microRNA Expression Pattern

Among the group of BD and control salivary RNA samples, we recruited samples for molecular analysis of Pri-miRNAs gene expression. The ACTB gene related to β-actin was used as the housekeeping gene.

We designed PCR oligos using the same in silico procedures previously described for absolute quantitation of different bacterial taxa. The RT-qPCR procedure was performed following the method described by Maxia et al., 2023 [88]. Table 2 shows the main parameters of oligonucleotides used in RT-qPCR. Relative expression was performed by using the 2^−∆∆Ct^ method by Livak et al. [89].

### 2.6. Statistical Analysis

For each sample, three distinct biological replicas were obtained, and quantitative data were expressed as means ± SDs. Changes in gene expression above 2 or below 0.5 were considered significant. Statistically significant differences for the exact determination among cases and control samples were determined using Fisher’s exact test to substantiate a significant difference between the means of two specific groups; in this case, statistical analysis was performed by using social science statistics software (version 2025, https://www.socscistatistics.com/, accessed on 20 January 2025). Given the small sample and non-normal distributions, we prioritised exact and non-parametric procedures, and we report two-sided *p*-values with a significance threshold of *p* = 0.05. No formal multiplicity adjustment, which increases the risk of type I error, was applied owing to the exploratory scope of the study; this is explicitly acknowledged in the limitations. Where informative, we emphasise the direction and magnitude of effects rather than dichotomous significance. Data were collected anonymously using an ID number for each person recruited. A dedicated database was created. For continuous variables, we applied the paired *t*-test when the normality assumption was met and verified the results using the Shapiro–Wilk test; we used the Wilcoxon signed-rank test otherwise. For categorical variables, we chose to replace the Chi-square test with McNemar’s test, which accounts for the dependence between paired observations. In addition, the Bonferroni multiple comparison test for molecular analysis was performed (α = 0.05), using GraphPad InStat software ver. 3.10 (San Diego, CA, USA).

## 3. Results

### 3.1. Bacterial Taxonomic Profiles Between Cases and Controls

**Figure 1:**
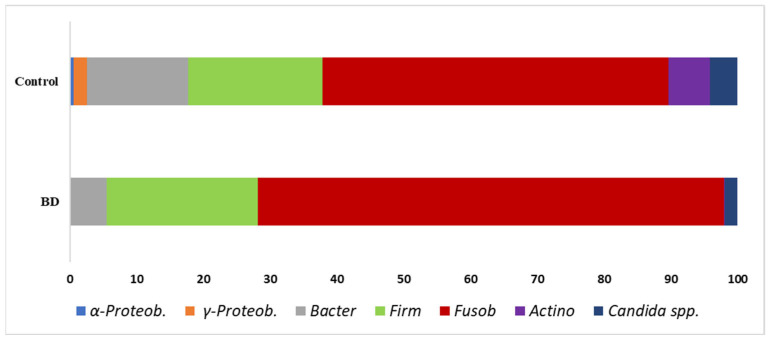
Main oral bacterial groups analysed in this work: Graph represents the percentage of the mean of all values. Individual subject values are overlaid on the bar plots to illustrate the full distribution of data (BD, n = 25; Controls, n = 46); a clear representation is shown in Figure 2.

The data obtained from samples of BD patients and controls using real-time qPCR are shown as taxa percentages in Figure 1; these represent an overview of the percentage relationships between the main bacterial phyla/genera in the oral cavity.

**Figure 2:**
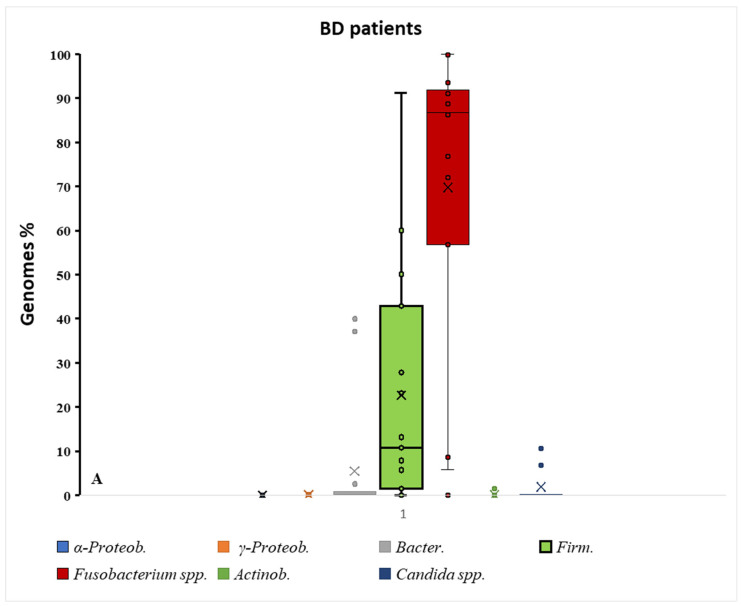

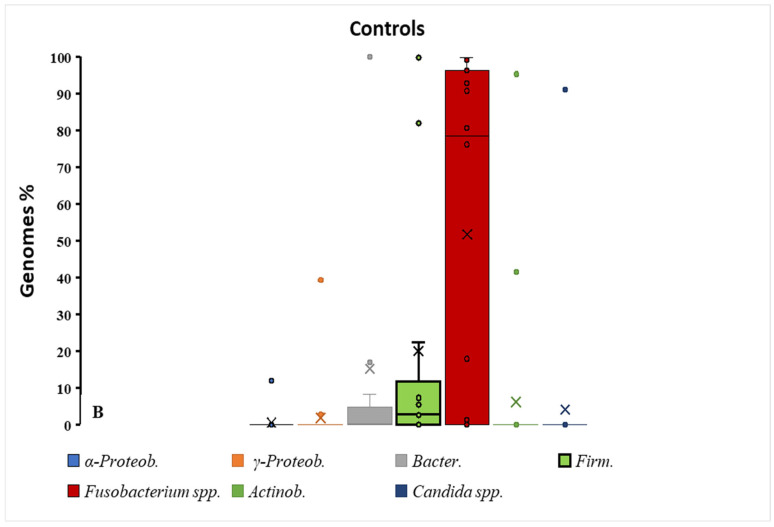
Box–dot plot distribution of different bacterial taxa % in BD and control groups. As shown in graph (**B**), a major distribution of percentages is observed for *Fusobacterium* spp. in the healthy control group vs. BD patients (**A**). The contemporaneity of Firmicutes reduction in this group indicates a major event of dysbiosis in healthy subjects; × = mean and horizontal line (-) in the box represent median value.

The taxonomic profile revealed in Figure 2 shows a substantial difference between mean and median % values. However, if we screen the results affected by extreme values according to the mean, the three main target bacterial groups that showed a significant difference were α-Proteobacteria, γ-Proteobacteria, and Actinobacteria; this aspect will be discussed in the next chapter. Because several taxa displayed right-skewed distributions with extreme values, medians provide a more robust summary than means. Importantly, the direction of the case–control differences was consistent across both central tendency measures. Statistical contrasts were conducted with non-parametric tests, which are less sensitive to outliers in small samples.

The specificity of amplification was supported by single-peak melting curves and product sizes matching the theoretical amplicons, ensuring that the observed taxa differences reflected true quantitative variation.

An elevated oral F/B ratio in BD should be interpreted as a marker of oral biotic status rather than a diagnostic signature. It reflects a relative increase in Firmicutes and/or depletion in Bacteroidetes and, in the oral niche, does not imply causality nor disorder specificity.

The F/B ratio was about 4/1 for BD subjects. Given the small sample size and skewed distributions, medians were prioritised as the most robust measure of central tendency, while means are reported descriptively. No formal adjustment for multiple testing (e.g., FDR or Bonferroni correction) was applied, consistent with the exploratory nature of this pilot study; this increases the risk of type I error and is acknowledged in the limitations. 

### 3.2. miRNA 155 and 146a Expression Patterns

**Figure 3:**
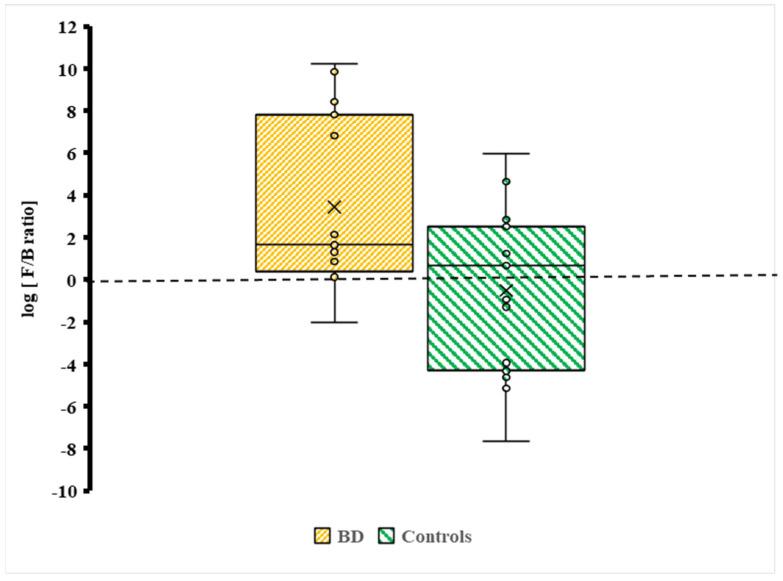
Distribution of the Firmicutes/Bacteroidetes (F/B) log ratio between BD cases and controls. Individual data points are plotted to display variability across participants (BD, n = 25; Controls, n = 46). The brown box represents BD cases, and the green box represents controls. × = mean and horizontal line (-) in the box represent median value.

Figure 3 demonstrates that pri-miRNAs evaluated at *p* > 0.05 showed no significant differences between the two patient cohorts. At the same time, the results showed a high dispersion since the SD was ± 2 for cases and ± 1.7 for controls. This behaviour may indicate that other factors, such as habits or health conditions of many subjects, are altering the equilibrium of the oral mucosal tissues. For both pri-miR-146a and pri-miR-155, melting-curve profiles confirmed single products of the expected size, with no amplification in negative controls. The lack of between-group differences for pri-miR-146a and pri-miR-155, together with the wide inter-individual variability, suggests that these transcripts may be strongly modulated by host factors (e.g., oral hygiene, smoking, medications, and diet) and pre-analytical variability. Accordingly, the negative finding should not be taken as evidence against a role of miRNAs in BD but rather as a call for more controlled, adequately powered studies.

## 4. Discussion

Until now, the ideas and perspectives regarding bipolar disorder and human microbiota have been inserted into the gut–brain axis machinery [90,91,92,93,94]. Historically, the gut microbiota has been identified as the major bacterial community in the body, and it has been powerfully demonstrated that this microbiota can modulate various mood statuses and host habits, for example, alimentary habits [90,95,96,97,98,99]. Research has identified bacteria, such as Bifidobacterium and Faecalibacterium, as modulators of brain–gut interactions through their impact on short-chain fatty acid (SCFA) production from dietary fibres [91,100,101]. These short molecules can interact with the nervous system through different mechanisms: (i) as anti-neuroinflammatory agents [102,103,104], (ii) neurogenesis modulation [105,106], and (iii) modulation of the pattern of some neurotransmitters, such as serotonin and GABA [107,108]. For this reason, gut microbial eubiosis is strictly necessary to achieve gut–brain biological equilibrium; on the downside, recurrent dysbiosis events in the gut could modify the bacterial metabolites involved in brain signalling and, therefore, the mood condition in the patient [102,109,110,111,112]. Our results should be considered preliminary and interpreted with caution due to the modest sample size and the exploratory, cross-sectional design. The observed taxa differences are associative signals and cannot be used to infer causality or diagnostic accuracy at this stage.

### The Crucial Role of Oral Microbiota

Our aim was to examine whether oral microflora contribute to gut eubiosis or dysbiosis. Evidence suggests that oral pathobionts can alter the gut microbiota, with studies showing that *Porphyromonas gingivalis* promotes systemic and neurodegenerative disorders such as Parkinson’s and Alzheimer’s.

**Figure 4:**
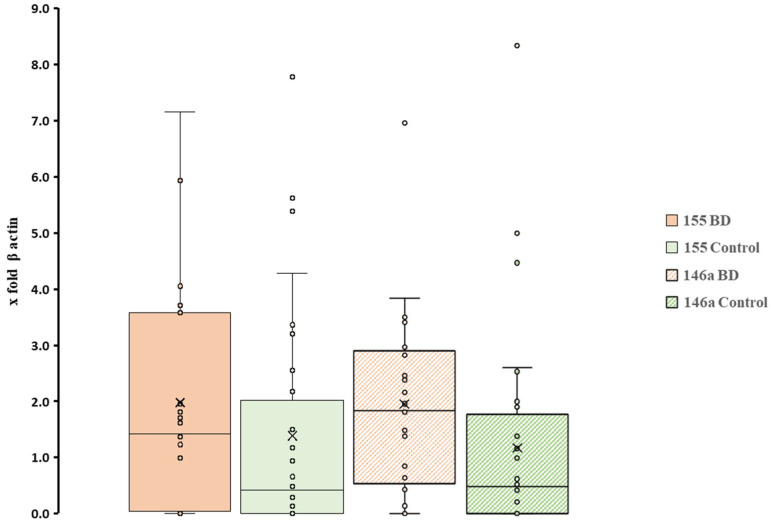
Box plot representation of expression patterns of two miRNAs between BD patients and controls. All individual sample values are displayed to illustrate variability between subjects (n = 25 BD; n = 46 controls; in all cases *p* > 0.05). × = mean and horizontal line (-) in the box represent median value.

**Figure 5:**
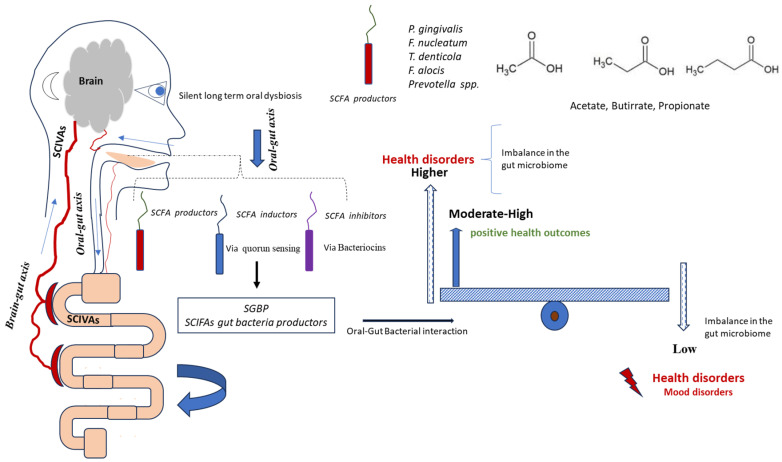
Schematic representation of oral bacteria and SCFA modulation for gut microbiota. The figure illustrates the potential interactions whereby bacteria from the oral cavity may influence gut microbiota composition and systemic SCFA levels, either positively or negatively. This conceptual model highlights the hypothesised roles of α-Proteobacteria, γ-Proteobacteria, and Actinobacteria in SCFA production and gut microbial balance. n = 25 BD; n = 46 controls.

A potential link between oral bacteria, gut microbiota, and mood disorders is theoretically plausible, though current data remain inconsistent. SCFA production illustrates this duality: locally, high SCFA levels can promote dysbiosis and oral disease, whereas in the gut, SCFAs exert systemic benefits, including anti-inflammatory and metabolic effects. These divergent outcomes arise from differences in mucosal structure, with the gut epithelium being adapted to absorb and utilise SCFAs. Oral microbes may, therefore, act as direct producers, inducers, or inhibitors of gut SCFA metabolism (Figure 4 and Figure 5).

The figure, presented in schematic mode, illustrates the complexity of oral–gut interactions. Bacteria from the oral cavity can interact positively or negatively with SCFAs, leading to changes in the amount of fatty acids in the gut and blood. Considering the results shown in Figure 1 and Figure 2, the significant variations in bacterial taxa are observed in three main microbial groups, already cited in the Section 3: α-Proteobacteria, γ-Proteobacteria, and Actinobacteria. Specifically, we can observe a significant reduction in these taxa in the oral cavity of BD patients.

These taxa contain more species of fatty acid-producing bacteria, such as those in the phylum Actinobacteria and the phylum Proteobacteria [113]. However, it is plausible that changes in the levels of SCFAs in the gut might be caused by certain bacteria that either work against or together with SCFA-producing bacteria [114,115,116,117,118]. The production of bacteriocins against gut bacterial SCFA producers could represent an interesting antagonistic mechanism [119]. The reduction in these phyla in BD patients may contribute to altered short-chain fatty acid (SCFA) production in the gut, possibly through modulation of biofilm networks. Oral bacteria, via quorum sensing mechanisms, could favour biofilm communities less associated with SCFA generation [119,120].

Finally, we found that the levels of pri-miRNAs 155 and 146a were similar in both the cases and control subjects, with no significant differences between the two groups. This might suggest that these genomic regulators in saliva are not strongly linked to BD conditions and could be influenced by other habits of the host. In another, already published work that uses used the amounts of volatile sulphur compounds in the breath of BD patients, which is related to dysbiotic status, the authors found a moderate production of these bacterial metabolites in these patients which suggests that the change in the oral microbiome able to drive the association between gut microflora and mood disorders could often be due to silent dysbiotic processes [36,121,122,123]. Second, while multiple statistical contrasts were performed, no correction for multiplicity was applied, in keeping with the hypothesis-generating scope of this work. These findings should not be regarded as evidence of biomarker potential at this stage but rather as hypothesis-generating signals that warrant confirmation in larger cohorts. Mechanistic studies, ideally including direct measurement of microbial metabolites such as SCFAs, as well as longitudinal designs accounting for medication exposure, will be essential to validating and contextualising these associations.

Limitations: Several limitations should be acknowledged when interpreting the present findings. First, the relatively small sample size, particularly in the bipolar disorder group, inevitably reduces statistical power and may limit the generalisability of the results, especially for highly variable biomarkers such as salivary pri-miRNAs. Second, salivary RNA is inherently subject to variability in quality and yield, which, although partially controlled through the use of a stable housekeeping gene (ACTB), may still have influenced the quantitative results and contributed to the observed heterogeneity. Furthermore, while primer design and validation were performed according to standard in silico and in vitro procedures, we did not conduct a comprehensive MIQE-compliant evaluation (e.g., the systematic reporting of amplification efficiencies, dynamic ranges, and reference gene validation). This omission should be regarded as a limitation, and future studies should implement full MIQE guidelines to maximise reproducibility and comparability across laboratories. Third, this study employed a targeted qPCR-based approach designed to quantify selected oral bacterial taxa; as such, it does not allow for the calculation of comprehensive microbial diversity indices (e.g., Shannon and Simpson) or ordination analyses (PCA/PCoA), which are standard in sequencing-based microbiome studies. Future investigations integrating 16S rRNA sequencing or metagenomic profiling will be necessary to fully characterise ecological diversity in this population. Furthermore, while multiple statistical contrasts were performed, no correction for multiplicity was applied, in keeping with the hypothesis-generating scope of this work. Therefore, the observed differences should be interpreted cautiously, and replication in larger, adequately powered samples will be essential. In addition, medication exposure was not systematically assessed. Psychotropic agents such as mood stabilisers, antipsychotics, and antidepressants, as well as antibiotics and other treatments, may substantially influence both oral microbiota and pri-miRNA expression. The absence of structured data on pharmacological profiles represents a relevant limitation of this pilot study and should be rigorously addressed in future research.

Finally, information on potential confounders such as smoking habits, oral hygiene, recent antibiotic exposure, and psychotropic medication use was not systematically available; thus, residual confounding cannot be excluded. Future studies should aim to incorporate detailed assessments of these factors and, where possible, adjust for them analytically to refine the observed associations.

## 5. Conclusions

In conclusion, this exploratory study identifies case–control differences in selected oral bacterial taxa among individuals with BD; however, these signals are preliminary and not sufficient for biomarker designation. Pri-miR-146a and pri-miR-155 did not discriminate BD status in this cohort, likely reflecting substantial inter-individual variability. Future work should prioritise much larger, longitudinal designs with comprehensive microbial profiling and rigorous control of confounders to test the reproducibility and clinical relevance of these associations. In this work, we employed a microbiomics approach to investigate the variation in major oral bacterial taxa in the oral microbiota between BD patients and controls. The data, highly preliminary, suggest significant mean variations in α-Proteobacteria, γ-Proteobacteria, and Actinobacteria, a great group of bacterial phyla involved in different potential mechanisms able to modulate gut bacteria. One potential mechanism could involve the interaction between the oral microbiota and the community of short fatty acid-producing bacteria, which can either potentiate or repress specific effects. Different authors relate the SCFAs decrease to different mood disorders. Even if further studies are necessary to assess the potential role of oral bacterial indicators for BD risk, this approach could be an effective method for evaluating the risk associated with these health conditions.

## Figures and Tables

**Table 1:** PCR oligos used in the real-time PCR method.

Name	Sequence 5′-------------------------3′	Phylum/Genus	Accession No.	bp **
OG1001F	AAACTCAAAKGAATTGACGG	Universal *	NR_024570.1	175
OG1002R	YTCACRRCACGAGCTGAC			
OG1003F	CKAGTGTAGAGGTGAAATT	α-Proteobacteria *	U11021.1	247
OG1004R	CCCCGTCAATTCCTTTGAGTT			
OG1005F	TCGTCAGCTCGTGTYGTGA	γ-Proteobacteria *	NR_026078.1	154
OG1006R	CGTAAGGGCCATGATG			
OG1007F	CRAACAGGATTAGATACCCT	Bacteroidetes *	NR_074784.2	203
OG1008R	GGTAAGGTTCCTCGCGTAT			
OG1009F	TGAAACTYAAAGGAATTGACG	Firmicutes *	NR_042772.1	152
OG1010R	ACCATGCACCACCTGTC			
OG1011F	TACGCTGGGCTACACACGTGC	Fusobacterium	NR_074412.1	213
OG1012R	AACCAACTCTCGTGGTGTGAC			
OG1013F	TACGGCCGCAAGGCTA	Actinobacteria *	NR_181378.1	303
OG1014R	TCRTCCCCACCTTCCTCCG			
OG1015F	AAGCTCGTAGTTGAACCTTG	*Candida* spp.	NG_070791.1	216
OG1016R	ATGGTCCTAGAAACCAACAA			
OG33-2	CCAGCAGCCGCGGTA	*E. coli*	NR_024570.1	286
OG123-2	GACTACCRGGGTATCTAATC			

Legend: * = primers described in Bacchetti et al., 2014 [86]; ** = PCR amplicon length.

**Table 2:** RT-PCR oligos designed for pri-miRNA gene expression.

Name	Sequence 5′-------------------------3′	Gene	Accession No.	bp *
OG 650F	GCATGGGTCAGAAGG	ACTB	PQ040393.1	297
OG 650R	AGGCGTACAGGGATAG			
OG1017F	TTTACAGGGCTGGGACAG	MHH2 (146a)	LC685969.1	191
OG1017R	TCAGGATCTACTCTCTCCAGG			
OG1018F	AGGAAGGGGAAATCTGTG	MIR155HG (155)	NR_001458.3	210
OG1018R	TCATGCTTCTTTGTCATCCT			

Legend: * = nucleotides, PCR amplicon length.

## Data Availability

The data presented in this study are available upon request from the corresponding author. Due to privacy and ethical issues, the data are not publicly available.
